# Multifaceted role of matrix metalloproteinases (MMPs)

**DOI:** 10.3389/fmolb.2015.00019

**Published:** 2015-05-13

**Authors:** Divya Singh, Sanjeev K. Srivastava, Tapas K. Chaudhuri, Ghanshyam Upadhyay

**Affiliations:** ^1^Department of Biology, City College of New YorkNew York, NY, USA; ^2^Cellular Immunology Laboratory, Department of Zoology, University of North BengalSiliguri, India

**Keywords:** MMP2, MMP9, neurodegenerative disorders, Japanese encephalitis, glaucoma

## Abstract

Matrix metalloproteinases (MMPs), a large family of calcium-dependent zinc-containing endopeptidases, are involved in the tissue remodeling and degradation of the extracellular matrix. MMPs are widely distributed in the brain and regulate various processes including microglial activation, inflammation, dopaminergic apoptosis, blood-brain barrier disruption, and modulation of α-synuclein pathology. High expression of MMPs is well documented in various neurological disorders including Parkinson's disease (PD), Alzheimer's disease (AD), Japanese encephalitis (JE), and Glaucoma. Although potentially critical, the role of MMPs in neuronal disorders is under-investigated. The present review summarizes the role of MMPs in neurodegeneration with a particular emphasis on PD, AD, JE, and Glaucoma.

## Introduction

Matrix metalloproteinases (MMPs) are described as calcium-dependent zinc endopeptidases. The roles of MMPs have been demonstrated in extracellular matrix (ECM) degradation and remodeling of tissues. MMPs specifically act on the different segments of ECM like collagen, gelatin, and elastin. MMPs were first discovered in 1962, and interstitial collagenase protein that cleaves collagen was initially isolated in 1968 (Gross and Lapiere, 1962; Eisen et al., 1968). MMPs are classified into four primary subgroups on the premise of domain structure: collagenases, gelatinases, stromelysins, and film sort (MT)-MMPs. Collagenases degrade triple helical fibrillary collagens, which are the fundamental segments of bone and ligament. Gelatinases (MMP-2 and MMP-9) are included in angiogenesis and neurogenesis by corrupting basal lamina molecules and subsequently leading to the cell death. Stromelysins (MMP-3, MMP-10, MMP-11, and MMP-7) are small proteases that degrade segments of the extracellular matrix. MT-MMPs activate a few proteases and development components at the cell surface. As of late, an expanding measure of proof proposes that MMPs may assume an essential part in the pathogenesis of various neurodegenerative disorders (Forsyth et al., 1999; Lorenzl et al., 2002, 2003; Yong et al., 2007; Candelario-Jalil et al., 2009; Choi et al., 2011; Shin et al., 2012). Although potentially imperative, the role of MMPs in neuronal disorders is under-investigated. In this brief review, we have made an attempt to shed a light on the role of MMPs in neurological disorders with a particular emphasis on Parkinson's disease, Alzheimer's disease, Glaucoma, and Japanese encephalitis.

## MMPs in nervous system

The mammalian CNS contains more than 10 different types of MMPs that can be detected at mRNA and protein levels (Pagenstecher et al., 1998; Ulrich et al., 2005; Fujioka et al., 2012). The studies have demonstrated that MMPs participate in the neuronal development and have ability to change the response to injury and neurological disease (Pagenstecher et al., 1998; Yong et al., 2001; Ulrich et al., 2005; Yong, 2005; Fujioka et al., 2012). Their expression profile varies according to the developmental program. Analysis has shown a developmental regulation of several of several MMPs including MMP-2, -9, -11, -12, -13, -14, -15, and -24, whereas certain MMPs for example MMP-3, -7, and -10 don't respond to these signals and remain unchanged during neuronal development (Pagenstecher et al., 1998; Ulrich et al., 2005; Yong, 2005; Fujioka et al., 2012). Remarkably, the extensive research is done on the role of two secreted MMPs, MMP-2, and MMP-9 since they are detected relatively easily in central nervous system (CNS). Both MMP-2 and MMP-9 are mostly detected in the cortex and cerebellum. MMP-9 is also detected in the hippocampus suggesting its critical role in neuronal biology (Pagenstecher et al., 1998; Ulrich et al., 2005; Yong, 2005; Fujioka et al., 2012). Genetic ablation of specific MMPs altogether influences the injury and pathology, therefore, essentially mediates neuronal disorders, including learning and memory (van Den Steen et al., 2002; Ethell and Ethell, 2007; Hu et al., 2007). In addition, MMPs likely intercede the structural modifications of dendritic spines and also axon/dendrite structures (Ethell and Ethell, 2007; Hu et al., 2007).

## Role of MMPs in parkinson's disease

Parkinson's malady (PD) is the most widely recognized neurodegenerative disorder described by motor symptoms including resting tremor, unbending nature, bradykinesia, and postural unsteadiness due to specific loss of dopaminergic neurons in the substantia nigra pars compacta. It is likewise a late onset disease and often noticed in the individuals over the age of 50 years. It is widely recognized nervous system disorder of the elderly. Hereditary, ecological, and age-related variables assume decisive parts in the loss of dopaminergic neurons and glial cells that may prompt Parkinson's disease (Lindvall and Kokaia, 2006).

It is now well established that the expression of several MMPs and their inhibitors are altered in the substantia nigra (SN) of brain of patient with PD proposing the urgent need for further investigation of the role of MMPs in PD (Lorenzl et al., 2002). MMPs have been recommended to contribute to the pathophysiology of PD by microglial activation, inflammation, dopaminergic apoptosis, blood-brain barrier (BBB) disruption and modulation of α-synuclein pathology by cleavage (Kim et al., 2005; Choi et al., 2008; Joo et al., 2010; Kim and Hwang, 2011). MMP-3, a zinc-dependent proteolytic enzyme that is broadly dispersed in the brain and can activate select pro-MMPs along with the cleavage of cell adhesion molecules, chemokines, and cytokines, is likewise reported in PD pathogenesis, especially in DA neuronal death, BBB damage, and neuroinflammation (Kim and Joh, 2006; Kim and Hwang, 2011; Van Hove et al., 2012a,b; Chung et al., 2013). The role of MMP-9 has also been implicated in PD, and its higher promoter activity due to C(-1562)T polymorphism was observed in a recent study (He et al., 2013). Furthermore, MMP1 has also been shown to be involved in PD pathogenesis in a case-control study by Gupta et al. demonstrating lower levels of MMP-1 in PD patients contrasted with the healthy controls. The study further stated a poor but statistically significant correlation between MMP-1 level and disease duration (Gupta et al., 2013).

## Role of MMPs in alzheimer's disease

Alzheimer's disease (AD) is characterized by spasms of the neurons leading to the loss of memory and behavioral changes. It is a late-onset disease, and manifestations show up mostly after the age of 60 years resulting in the progressive loss of memory and speculation abilities, and inevitably the patients can't perform even easiest assignments. AD is also suggested to be the major cause of dementia (Upadhyay et al., 2014). Estimates vary, but experts suggest that as many as 5.1 million Americans may have Alzheimer's disease (Upadhyay et al., 2014). Both hereditary and environmental components add to AD pathogenesis and an exclusion criterion (by eliminating the likelihood of different reasons for dementia) is taken into account alongside considering significant manifestations of AD for its diagnosis (Upadhyay et al., 2014).

Neuropathological hallmarks of Alzheimer's disease include neuronal tangles and amyloid plaques (Laferla et al., 2007; Rosenberg, 2009). Deposition of improperly processed amyloid is thought to be a main factor in the pathophysiology of AD. The role of MMPs in CNS pathology is now well known and different studies have demonstrated the significance of MMPs for the pathophysiological process amid AD. These MMPs are essentially included in the formation and clearance of the amyloid-β peptides (Aβ) and discovered to be induced by the amyloid molecules in blood vessels, astrocytes, and microglia (Yan et al., 2006; Yin et al., 2006; Walsh et al., 2007). The inflammatory response, such as activation of microglia and astrocytes in response to the deposition of Aβ in tissues around the plaques, might contribute to neuronal death in AD (Selkoe, 2008). The increase in expression of MMPs in brain tissues and blood of patients with AD is probably part of the inflammatory response. Furthermore, growing body of knowledge suggests the dual role of MMPs in AD pathogenesis. At one hand it directly degrade Aβ resulting in reduction in Aβ deposit (Yan et al., 2006; Miners et al., 2008); then again, these could contribute to brain parenchymal destruction when induced by Aβ in microglia, astrocytes or vascular smooth muscle cells. These data clearly indicate the involvement of MMPs in the pathogenesis of AD.

## Role of MMPs in japanese encephalitis

Japanese encephalitis (JE) that commonly affects children is caused by a single-stranded RNA virus, JE virus (JEV) results in severe neurological disorders (Kalita et al., 2007; Misra et al., 2009). It infects the central nervous system leading to acute encephalitis occurring in a broad spectrum of the Asian subcontinent (Kumar et al., 2009a). The transmission of JEV in humans occurs mostly by mosquito vector (Culex tritaeniorhynchus). JEV infection causes severe damage to neurons in the various parts of the brain (Kumar et al., 2009a). Additionally, JE infection has been reported to impair the balanced dynamic actions of oxidant and antioxidant systems in rats (Kumar et al., 2009b).

Although, the exact mechanism of neuronal cell death in JE is unclear, some studies established the role of MMPs in neuronal cell death. A recent study suggests that during JEV infection, the expression of MMPs (MMP-2, -7, -9) and TIMPs (TIMP-1 and -3) are greatly increased and can contribute to the disease severity in CNS (Shukla et al., 2012). Furthermore, It has been shown that the induction of MMP-9 is most likely due to AP-1 activation triggered by ROS/c-Src/PDGFR/PI3K/Akt/MAPKs pathway following JEV infection (Tung et al., 2010; Yang et al., 2012). Shukla et al. demonstrated higher concentrations of MMP-2, TIMP-2, and TIMP-3 in cerebrospinal fluid (CSF) and serum of children with JEV as compared with disease control (DC) (Shukla et al., 2013). They also observed a higher concentration of MMP-9 and MMP-7 in the serum of JEV patients when compared with DC and healthy control but not in CSF (Shukla et al., 2013).

## Role of MMPs in glaucoma

Glaucoma, a neurodegenerative disease, is a group of ocular disorders with multi-factorial etiology. It is the second leading cause of blindness after cataract in the world. Over 60 million people are affected worldwide while more than 2 million individuals are affected in the United States. Mainly it occurs in two forms- open angle glaucoma and closed angle glaucoma. Open angle glaucoma (also known as primary open angle glaucoma) is the commonest form of glaucoma where retinal ganglion cells (RGCs) degeneration progress slowly without showing any symptom at early stage while vision loss occurs quickly in later form of glaucoma. The most prevalent and major risk factor is elevated intraocular pressure (IOP) is the primary parameter to be used in the diagnosis of disease. Glaucoma is associated with optic nerve degeneration and RGCs death by apoptosis leading to visual field loss (Quigley, 1999; Tatton et al., 2001; Halpern and Grosskreutz, 2002). Several environmental, genetic and age-related factors contribute to the onset of the disease (Leske et al., 2007; Calkins and Horner, 2012).

Despite several efforts, the mechanism of RGC and optic nerve degeneration is not clearly understood. However, some studies have been established the role of MMPs in the pathophysiology of glaucoma. MMPs, major matrix degrading enzymes are involved in remodeling of ECM of the trabecular meshwork to maintain stable aqueous humor outflow resistance and IOP (De Groef et al., 2013). Besides the anterior segment of the eyes, MMPs are also involved in the regulation of retinal cell death. MMP-9 is reported to promote the RGC death by abolishing integrin-mediated survival signaling pathways via laminin degradation (Guo et al., 2005; Halfter et al., 2005; Santos et al., 2012). The role of MMPs in the regulation of outflow resistance has also been studied in steroid-induced glaucoma (SIG), a form of glaucoma that resembles with primary open angle glaucoma. SIG is caused by the potent use of glucocorticoids which results in a decreased trabecular outflow leading to increased IOP (Kumar et al., 2013b). The up-regulation of several MMPs specifically MMP-2, MMP-9, and MMP-13 are reported in steroid induced mice model of glaucoma (Kumar et al., 2013a). In another study, mRNA expression of MMP-1 (but not MMP-2, MMP-9, and MMP-13) was found to be significantly up-regulated in steroid induced sheep model of glaucoma. (Gerometta et al., 2013).

## Conclusion

Our understanding about the role of MMPs in neurological disorders including Glaucoma and JE has increased enormously during past few years. Nevertheless, the imperative role of MMPs in the pathogenesis of the elderly neurological diseases, such as AD, PD and so forth needs further and extensive investigation. Since the role of MMPs in the regulation of the pathological alterations in the blood vessels and ECM around cells is unfolding, the need of the hour is to investigate these in the clinics. It is remarkable that these processes determine the fate of cell survival and permeability of BBB. Targeting these events could be of great therapeutic value, and it is necessary to explore the role of MMPs more widely and wisely in order to develop new potential strategies for the cure of neurological disorders.

### Conflict of interest statement

The authors declare that the research was conducted in the absence of any commercial or financial relationships that could be construed as a potential conflict of interest.
